# Effect of strain-specific maternally-derived antibodies on influenza A virus infection dynamics in nursery pigs

**DOI:** 10.1371/journal.pone.0210700

**Published:** 2019-01-14

**Authors:** Fabian Orlando Chamba Pardo, Spencer Wayne, Marie Rene Culhane, Andres Perez, Matthew Allerson, Montserrat Torremorell

**Affiliations:** 1 Veterinary Population Medicine Department, University of Minnesota, St. Paul, MN, United States of America; 2 Health Services, Pipestone Veterinary Services, Pipestone, MN, United States of America; 3 Health and Research Division, Holden Farms Inc., Northfield, MN, United States of America; University of Georgia, UNITED STATES

## Abstract

Reducing the number of influenza A virus (IAV) infected pigs at weaning is critical to minimize IAV spread to other farms. Sow vaccination is a common measure to reduce influenza levels at weaning. However, the impact of maternally-derived antibodies on IAV infection dynamics in growing pigs is poorly understood. We evaluated the effect of maternally-derived antibodies at weaning on IAV prevalence at weaning, time of influenza infection, number of weeks that pigs tested IAV positive, and estimated quantity of IAV in nursery pigs. We evaluated 301 pigs within 10 cohorts for their influenza serological (seroprevalence estimated by hemagglutination inhibition (HI) test) and virological (prevalence) status. Nasal swabs were collected weekly and pigs were bled 3 times throughout the nursery period. There was significant variability in influenza seroprevalence, HI titers and influenza prevalence after weaning. Increase in influenza seroprevalence at weaning was associated with low influenza prevalence at weaning and delayed time to IAV infection throughout the nursery. Piglets with IAV HI titers of 40 or higher at weaning were also less likely to test IAV positive at weaning, took longer to become infected, tested IAV RT-PCR positive for fewer weeks, and had higher IAV RT-PCR cycle threshold values compared to piglets with HI titers less than 40. Our findings suggest that sow vaccination or infection status that results in high levels of IAV strain-specific maternally-derived antibodies may help to reduce IAV circulation in both suckling and nursery pigs.

## Introduction

Influenza A virus (IAV) is a primary cause of acute respiratory disease in pigs and it is also part of the porcine respiratory disease complex (PRDC), which includes other pathogens such as porcine reproductive and respiratory syndrome virus (PRRSV), porcine circovirus 2 (PCV2), and *Mycoplasma hyopneumoniae* [1]. IAV infection affects the performance of pigs by increasing feed conversion [2] and mortality [3], decreasing body weight gain [4] and reducing semen quality of boars [5]. IAV in pigs also represents a threat to public health since it is a zoonosis with pandemic potential.

Influenza is widespread in US pigs and breed-to-wean (BTW) pig farms play a central role in the spread of IAV across geographical regions [6–9]. Suckling pigs maintain, diversify and transmit IAV when moved to other farms [10–13]. Commonly, pigs are weaned at 21 days of age and are moved to distant locations to grow to market. The significant IAV genetic diversity in pigs is a result not only from pig movements but also due to the rapid mutation rate of the virus (genetic drift) [14–17] and the introduction of IAV from humans (reverse zoonosis) [18–23] and birds [24–26], which facilitates the emergence of novel reassorted strains (genetic shift). H1N1, H1N2 and H3N2 are the most common subtypes found in pigs and the introduction of gene segments from other species into a pool of endemic viruses has resulted in a complex landscape of IAV in pigs. Indeed, currently there are 16 genetically and antigenically distinct H1 clades (alpha, beta, gamma, gamma 2, delta 1a, delta 1b, delta 2 and pandemic 2009) [15, 17, 27] or H3 clusters (IV A-F, human-like 2011 and human-like 2016) [9, 14, 28, 29] of IAV co-circulating in US pigs. This broad genetic diversity and the common co-circulation of several clades within a farm or production system represents a critical hurdle for vaccines to induce cross-protective immunity effective against genetically diverse strains [30, 31].

Sow vaccination is the main measure to control influenza in BTW farms [32–35]. Sow vaccination helps protecting the herd from severe clinical disease and enhances the transfer of maternally-derived antibodies (MDA) from sows to piglets through colostrum. MDA protect piglets from clinical disease shortly after weaning [36–39] and strain-specific MDA can also decrease IAV transmission in weaned pigs [40]. However, although the use of IAV vaccines in BTW farms is common, IAV still circulates in pigs likely due to the limitations for generating sufficient levels of immunity against the multiple strains co-circulating [11, 41].

In addition, pigs have complex IAV infection dynamics, such as recurrent infections, multiple waves of infection, co-circulation of genetically distinct viruses and frequent reassortment events observed in growing-finishing pigs [42–49]. However, quantitative knowledge on the factors that are associated with the complex IAV infection dynamics in nursery pigs is limited. Also, there is limited information about how IAV MDA may impact infection dynamics in nursery pigs. Filling this knowledge-gap is critical to guide successful intervention strategies for BTW farms that aim to reduce IAV circulation in pigs before and after weaning. In our study, we evaluated the effect of IAV seroprevalence and individual MDA levels at weaning on IAV prevalence at weaning, time to IAV infection after weaning, number of weeks that pigs tested IAV RT-PCR positive, and IAV RT-PCR cycle threshold values in pigs after weaning. Results from our study contributed to determine whether sow interventions in the breeding herd have a direct benefit in piglets at weaning and throughout the growing period in the nursery.

## Materials and methods

### Ethics statement

This study was carried out after the protocol was approved by the Institutional Animal Care and Use Committee (Protocol Number: 1510-33054A) and the Institutional Biosafety Committee (Protocol Number: 1508-32918H) of the University of Minnesota. The participating producers provided written consent to conduct the study in their farms. All samples were collected by trained veterinarians and researchers. Pigs were raised indoors in conventional mechanically ventilated nursery barns with climate control. Pigs had ad libitum access to fresh food and water and farmers monitored the health of the pigs twice daily to detect and treat any sick pig. Pigs were cared according to recommendations by the herd veterinarian which included antipyretic and antibiotic treatments on as needed basis.

### Study population and design

Piglets (n = 301) were identified at weaning and tested during the growing period in the nursery which included piglets from 3 to 10 weeks of age. Five batches of weaned piglets were placed into 2 separate nursery farms. Pigs in each batch were divided into 2 rooms for a total of 10 cohorts. Approximately 30 pigs within each cohort were selected and pigs were placed in a randomly selected pen within each room. Each nursery farm was a single-barn site with two rooms with all-in/all-out flow by site (all pigs entered and exited at once to facilitate cleaning and disinfection of the entire facility). Piglets were vaccinated at weaning against *Mycoplasma hyopneumoniae*, PCV2 and PRRSV (Ingelvac 3FLEX^®^ Vaccine, Boehringer Ingelheim Vetmedica, Inc., St. Joseph, MO).

Piglets originated from a single air-filtered 3,200 sow farm located in Minnesota with a history of testing IAV positive by RT-PCR in piglets prior to weaning for at least the last 2 months before the study began. The sow farm was located within 10 miles from the nursery farms and known to be negative for wild-type PRRSV strains but positive for *M*. *hyopneumoniae* and PCV2. Additionally, incoming replacement females (gilts) were vaccinated twice with an inactivated IAV commercial vaccine (FluSure XP^®^; Zoetis Inc, Parsippany, NJ).

The study was conducted from November 2015 to April 2016. During that time, sows were vaccinated against PRRSV using a modified-live vaccine (Ingelvac PRRS^®^ MLV vaccine, Boehringer Ingelheim Vetmedica, Inc., St. Joseph, MO) as part of their biannual vaccination against PRRSV. During 8 weeks within the study period (December 2015 to January 2016), sows were also vaccinated with 2 commercial IAV inactivated vaccines (Maxivac Excell 5.0^®^, Merck Animal Health, Madison, NJ; and FluSure XP^®^, Zoetis Inc, Parsippany, NJ) with cohorts 3a, 3b, 4a and 4b originating from vaccinated sows. There was no sow influenza vaccination before and after the described 8-week period during the study timeframe. Cohort details are explained in Table 1.

**Table 1:** Cohort information.

Cohort ID	Nursery farm ID	Nursery room ID	No. pigs/room	No. pens/room	No. pigs sampled	Sow IAV vaccination
**1a**	1	A	707	28	28	No
**1b**	1	B	707	28	28	No
**2a**	2	A	670	20	34	No
**2b**	2	B	670	20	31	No
**3a**	1	A	700	28	28	Yes
**3b**	1	B	699	28	27	Yes
**4a**	2	A	675	20	34	Yes
**4b**	2	B	675	20	34	Yes
**5a**	1	A	708	28	28	No
**5b**	1	B	709	28	29	No

### Sampling, testing and influenza characterization

Piglets in each cohort were ear-tagged, nasal swabbed and blood samples collected at weaning. Nasal swabs were collected weekly post-weaning until the end of the growing period in the nursery and blood samples collected at 3- and 6-weeks post-weaning. Nasal swabs were processed by suspending them in 2mL of viral transport media (Minimum Essential Media plus bovine serum albumin, antibiotics, antifungals and trypsin TCPK) and stored at -80°C until testing. Swabs were tested by a real-time RT-PCR targeting the IAV matrix gene [50].

Briefly, sample viral RNA was extracted using a MagMax^TM^—96 viral RNA extraction kit (Applied Biosystems, Foster City, CA) following manufacturer’s instructions using an automatized robotic extraction equipment: MagMax^TM^–Express 96 Deep well magnetic particle processor (Applied Biosystems, Foster City, CA). RT-PCR test reagents were from AgPath-ID one-step RT-PCR kit (Life Technologies, Grand Island, NY) and reactions were run in a 7500 Fast RT-PCR system (Life Technologies, Grand Island, NY). Thermal cycles for IAV detection were 10 min at 45°C, 10 min at 95°C, 45 cycles of 1 sec at 94°C and 30 sec at 60°C within a 25μL of total volume [50]. A sample was considered IAV rRT-PCR positive if the cycle threshold (ct) value was 37.5 or lower.

Additionally, two nasal swab samples with the lowest ct values in each cohort from weeks when IAV prevalence was the highest were selected for virus isolation in Madin-Darby Canine Kidney (MDCK) cells [51] and whole-genome sequenced (WGS) using the MiSeq Ilumina platform [52]. When IAV isolation was not possible, WGS was performed directly from selected nasal swab samples (cohorts 4a, 4b, 5a and 5b). Raw sequence reads were cleaned, trimmed and assembled against both H1 (FJ789832) and H3 (KC992248) reference strains obtained from the Influenza Virus Resource at the National Center for Biotechnology Information (NCBI) [53]. Hemagglutinin (HA) gene assembly was done using the Map to a Reference function in Geneious 8.1 software [54].

HA gene consensus sequences were annotated for completeness, functionality and subtype classification using the influenza virus sequence annotation tool (FLAN) [55]. Complete HA gene sequences were further characterized using BLAST tools and the global swine H1 clade was inferred using the swine H1 clade classification tool from the Influenza Research Database (IRD) [56].

HA gene sequences were aligned and translated into amino acid sequences to further analyze the amino acid similarities between viruses from the BTW farm, the studied cohorts and the IAV commercial vaccines used in sows. Alignment, amino acid translation and sequence comparisons were done using the ClustalW algorithm, Neighbor Joining method and a Jukes-Cantor substitution model in Geneious software [54]. H1 antigenic sites (Sa, Sb, Ca1, Ca2 and Cb) [57–61] were compared among the obtained sequences using the above described analysis and the Identify Sequence Features in Segments tool of the IRD [56].

Finally, blood samples were left at room temperature for serum separation and then refrigerated overnight. Samples were centrifuged for 10 min at 1500 rpm and 4°C. Sera then were stored at -20°C until testing. Sera were tested by hemagglutination inhibition (HI) assay using a representative and dominant IAV isolate (H1 delta 2) among all cohorts and BTW farm. Briefly, sera were pretreated with receptor destroying enzyme (RDE) and incubated at 37°C for 14 hours overnight. Incubated sera were hemadsorpted by adding a 20% turkey red blood cell solution, centrifuged, and stored at -20°C until titration. Two-fold serial dilutions from 1:10 to 1:640 were tested for each serum sample using an antigen solution containing 16 hemagglutination units (HAU) in 50uL and a 0.5% turkey red blood cell solution. Positive and negative controls were used in each plate to confirm the results obtained in plate. Plates were read manually by tilting them and visualizing inhibition of agglutination (presence of a mat of red blood cells in the bottom of the well) [62]. Additionally, 15 pigs in each cohort were selected according to their HI titers (half of the piglets within each HI titer) at weaning and serum samples tested by IAV NP ELISA (IDEXX Influenza A Ab Test, IDEXX Laboratories, Inc., Westbrook, Maine). A sample was considered positive if the S/N value was less than 0.6. ELISA testing was done according to the manufacturer’s protocol.

### Data analysis

Individual HI titer data was tabulated and classified as HI-positive or -negative using a reciprocal HI titer of 40 as the cutoff point (≥40 were considered positive). RT-PCR data were tabulated and classified as positive or negative based on a cycle threshold (ct) cutoff point of 37.5. Then, the percentage of IAV RT-PCR and HI serum antibody positive pigs over time in the nursery was calculated and values reported as prevalence and seroprevalence, respectively. Prevalence and seroprevalence data were summarized by week for each cohort.

Time to IAV infection was calculated by counting the number of weeks from weaning until a pig tested IAV RT-PCR positive. The number of weeks that a given pig tested IAV RT-PCR positive during the growing period in the nursery was calculated by counting the total number of weeks that a pig tested IAV RT-PCR positive for the entire nursery period. Because the real-time RT-PCR used in this study is semi-quantitative, the estimated quantity of IAV virus was approximated from the RT-PCR ct values, with the lowest ct values having the highest estimated virus quantity and ct values of >37.5 having estimated virus quantity approaching zero. For a given pig, the lowest ct value during the entire study was chosen for the median calculations by cohort. Median values of time to IAV infection, number of weeks that a pig tested IAV RT-PCR positive and lowest ct values were calculated for each cohort and for each reciprocal HI titer group using the dplyr package in R 3.4.2 statistical software [63].

To test the association between IAV seroprevalence of the cohort and HI titer at weaning with prevalence at weaning, time to IAV infection, number of weeks that pigs tested IAV RT-PCR positive and lowest ct values during the nursery period, a Spearman non-parametric correlation test was used in STATA 12^®^ statistical software (StataCorp, College Station, TX) [64]. Different regression models were used to test the association of HI titers at weaning with IAV RT-PCR positivity at weaning for each pig (logistic regressions), time to IAV infection and number of weeks that each pig tested IAV RT-PCR positive (Poison regressions), and lowest ct value of each pig during the growing period in the nursery (linear regressions). Regressions were modeled under generalized linear models (GLM) in the glimmix procedure of SAS 9.4^®^ statistical software (SAS Foundation, Cary, NC) [65]. Models were adjusted by nursery farm and season effects as fixed covariates. Best models were chosen based on goodness of fit criteria using the lowest Bayesian Information Criterion (BIC) and visual inspection of the residuals to check model assumptions accordingly.

## Results

A total of 301 piglets from 10 cohorts were tested from weaning to the end of the growing period in the nursery. Overall, there were 885 serum samples of which 28% had a reciprocal HI titer of 40 or higher. Moreover, 348 of 2176 (16%) nasal swabs were IAV RT-PCR positive. In total, 185 of 301 pigs (62%) tested IAV RT-PCR positive at least once during the study (S1 Table).

We obtained 20 IAV HA gene sequences including 19 from the cohorts and 1 from the BTW farm prior to the start of the study. We characterized the HA gene sequences as H1 delta 2 (1B.2.1) viruses having 99.4% nucleotide and 99.3% amino acid similarity among themselves. The H1 delta 2 HA gene sequences obtained during the study shared 94.3% and 94.8% amino acid similarity to the H1 delta 2 commercial vaccine strains used in the BTW farm and there were no amino acid changes in the H1 antigenic sites (Sa, Sb, Ca1, Ca2 and Cb).

**Fig 1:**
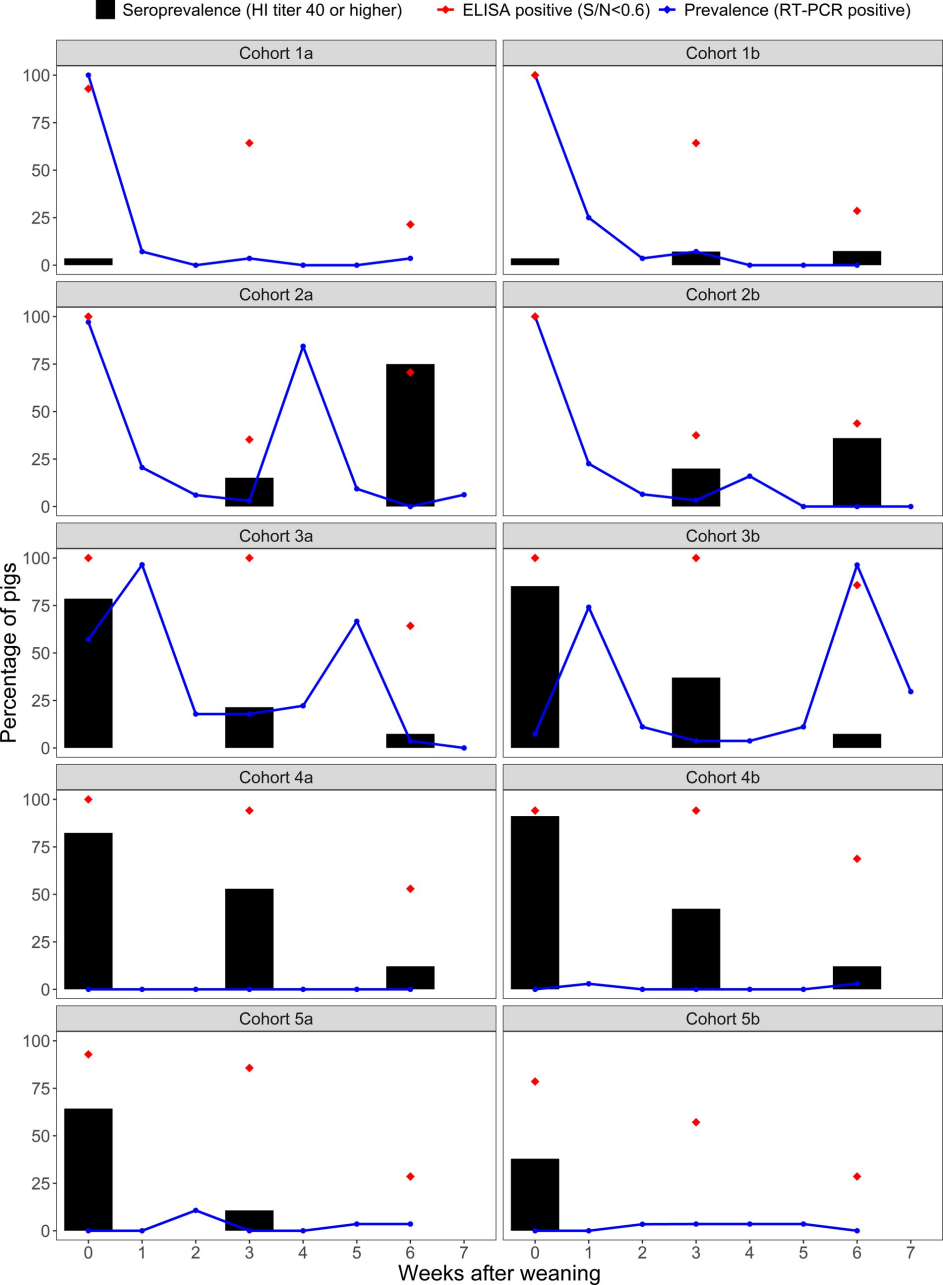
Influenza A virus prevalence and seroprevalence by cohort during the nursery period. Black bars are the seroprevalence (hemagglutination inhibition titer of 40 or higher), blue line is the prevalence (percentage of RT-PCR positives) and red dots is the percentage of NP ELISA positive pigs (S/N<0.6).

There was appreciable variability in prevalence and seroprevalence between cohorts over time (Fig 1). IAV prevalence at weaning changed from almost 100% in cohorts with low seroprevalence to about 0% in cohorts with high seroprevalence at weaning. Inversely, seroprevalence measured by HI titers against the H1N2 delta 2 IAV circulating farm strain at weaning changed over time from 0% in cohorts of piglets originating from non-vaccinated sows up to 80 and 90% in cohorts originating from vaccinated sows. Additionally, IAV prevalence during the growing period in the nursery at the cohort level varied. There were episodes of high prevalence of IAV infection at weaning (97–100%) with none or one recurrent infection after weaning to episodes of lower prevalence (~0%) at weaning with limited infection during the nursery, as shown in Fig 1.

IAV NP ELISA results summarized as percentage of ELISA positive pigs over time in each cohort are also shown in Fig 1 (red dots). Almost all pigs at weaning tested IAV NP ELISA positive and percentage of positive pigs declined over time or increased slightly after the second peak of infection, similar to the HI titers dynamics observed in most of the cohorts. However, cohorts 1a, 1b, 2a and 2b had a high percentage of NP ELISA positive pigs at weaning and pigs had low HI titers and high levels of IAV infection. In these cohorts, pigs did not appear to have HI strain-specific antibodies that could prevent IAV infection.

Per cohort, high seroprevalence at weaning was significantly associated with lower IAV prevalence at weaning (correlation value of -0.71) and it took a longer time, more than 6 weeks, to become IAV infected after weaning. The number of weeks that pigs tested IAV positive, and lowest ct values during the growing period in the nursery were not significantly associated with seroprevalence at weaning although, there was a numerical trend that reflected limited circulation of IAV after weaning. Table 2 illustrates the association between IAV seroprevalence at weaning and our calculated IAV infection data after weaning for each cohort.

**Table 2:** Cohort-level correlation of influenza A virus (IAV) seroprevalence at weaning with infection parameters in the nursery.

Cohort	Number of pigs	IAV seroprevalence (HI titer≥40) at weaning (%)	IAV prevalence at weaning (%)	Time to IAV infection (weeks) Median (IQR)	Number of IAV positive weeks Median (IQR)	Lowest RT-PCR ct value Median (IQR)
**1a**	28	4	100	0 (0)	1 (0)	27 (11)
**1b**	28	4	100	0 (0)	1 (1)	24 (8)
**2a**	34	0	97	0 (0)	2 (0)	31 (10)
**2b**	31	0	100	0 (0)	1 (1)	28 (9)
3a^*^	28	79	57	0 (1)	2.5 (1)	29 (8)
3b^*^	27	85	7	1 (0)	2 (1)	29 (7)
4a^*^	34	82	0	>6 (0)^a^	0 (0)	45 (0)
4b^*^	34	91	0	>6 (0)^a^	0 (0)	45 (0)
**5a**	28	64	0	>6 (0)^a^	0 (0)	45 (0)
**5b**	29	38	0	>6 (0)^a^	0 (0)	45 (0)
Spearman’s rho (r_s_)	-	Predictor	-0.71	0.66	-0.25	0.51
**p-value**	**-**	**Predictor**	**0.02**	**0.04**	**0.48**	**0.14**

HI, Hemagglutination inhibition assay; IQR, Interquartile range; RT-PCR, Real-time reverse transcriptase polymerase chain reaction; ct, RT-PCR cycle threshold.

* Cohorts 3a, 3b, 4a and 4b originated from vaccinated sows.

^a^ Time to IAV infection in cohorts 4 and 5 was assigned 6 weeks due to the termination of the study. We did not measure how many weeks those pigs remained IAV negative in the finishing sites.

HI titer group at weaning was significantly associated with infection dynamics after weaning as detailed in Table 3. According to their HI titer group at weaning, reciprocal HI titers of 40 or higher were significantly associated with lower IAV levels at weaning, longer time to IAV infection, fewer weeks that pigs tested IAV RT-PCR positive, and less estimated quantity of virus in groups of pigs during the growing period in the nursery.

**Table 3:** Correlation of influenza A virus (IAV) hemagglutination inhibition (HI) titer group at weaning with infection parameters in nursery pigs.

IAV HI titer group at weaning	Number of pigs	IAV prevalence at weaning (%)	Time to IAV infection (weeks) Median (IQR)	Number of IAV positive weeks Median (IQR)	Lowest RT-PCR ct value Median (IQR)
**<10**	81	79	0 (0)	1 (1)	31 (9)
**10**	51	90	0 (0)	1 (1)	29 (12)
**20**	34	38	1.5 (6)	1 (2)	35 (17)
**40**	53	11	6 (5)	0 (2)	45 (13)
**80**	34	15	6 (5)	0.5 (2)	41 (16)
**160**	21	10	6 (5)	0 (2)	45 (11)
**320**	19	11	6 (5)	0 (2)	45 (10)
**≥640**	8	0	6 (0)	0 (0)	45 (0)
Spearman’s rho (r_s_)	Predictor	-0.90	0.87	-0.85	0.85
**p-value**	**Predictor**	**0.002**	**0.005**	**0.008**	**0.008**

HI, Hemagglutination inhibition assay; IQR, Interquartile range; RT-PCR, Real-time reverse transcriptase polymerase chain reaction; ct, cycle threshold.

Individual HI titer at weaning was significantly associated with the IAV RT-PCR positive status during the growing period in the nursery as shown in Table 4. Pigs with a reciprocal HI titer of 40 or higher at weaning were less likely to test IAV RT-PCR positive at weaning, took longer to become IAV infected, tested IAV RT-PCR positive for fewer weeks and had higher ct values. We estimated a 5% probability of infection for pigs with reciprocal HI titers ≥ 40. Overall, high MDA levels at weaning decreased the likelihood of IAV infection and circulation at weaning and during the growing period in the nursery.

**Table 4:** Pig-level association of influenza A virus (IAV) hemagglutination inhibition titer at weaning with individual infection parameters during the nursery period.

IAV HI titer at weaning	Probability of a pig testing IAV positive at weaning	Mean time to IAV infection in weeks	Mean number of IAV positive weeks	Mean lowest RT-PCR ct value
**<10**	0.93^a^	0.07^a^	1.41^a^	30.2^a^
**10**	0.88^a^	0.05^a^	1.38^a^	30.2^a^
**20**	0.33^b^	0.13^b^	0.70^b^	33.9^b^
**40**	0.06^c^	0.17^c^	0.49^bc^	37.7^c^
**80**	0.05^c^	0.22^d^	0.45^bc^	39.2^cd^
**160**	0.05^c^	0.24^d^	0.34^c^	42.5^e^
**≥320**	0.05^c^	0.24^d^	0.30^c^	42.0^de^
**p-value**	**< .0001**	**< .0001**	**< .0001**	**< .0001**

HI, Hemagglutination inhibition assay; IQR, Interquartile range; RT-PCR, Real-time reverse transcriptase polymerase chain reaction; ct, cycle threshold. Different superscripts in each column mean statistically significant differences after adjusted pairwise comparisons (p-value<0.05).

## Discussion

Control of influenza in BTW farms is needed to minimize the spread of IAV across pig production systems and regions given that piglets infected at weaning covertly transmit IAV to other farms. Sow vaccination is the most common IAV control measure although the impact of MDA on infection after weaning is poorly understood. Our study showed that high levels of strain-specific MDA at weaning can decrease prevalence of IAV at weaning and more importantly, decrease the overall IAV circulation during the growing period in the nursery. Reducing IAV circulation at weaning should help decrease IAV transmission between farms.

Our study supports previous experimental work that demonstrated high MDA measured by strain-specific HI titers reduced significantly the transmission of IAV in weaned pigs [40]. It also supports case reports where IAV elimination was attempted using IAV strain-specific sow vaccination strategies that were successful in decreasing the detection of IAV in piglets at weaning for certain periods of time [34, 35]. Notably, our results help clarify the understanding of the effect of high levels of strain-specific MDA over IAV infection in the nursery phase under field conditions.

Our study was also similar to other studies that reported variable levels of IAV circulation and seroconversion in growing pigs and had one or more peaks of infection after weaning [37, 42–47]. In contrast, our results differed from studies where heterologous antibodies had been measured. In those studies, heterologous MDA had no effect on IAV transmission in weaned pigs, prolonged the infectious period and blocked the active immune response to infection [36, 39, 40, 66–69]. The immunological mechanism behind these latter observations is not well defined and B cell epitope masking has been proposed as one of the possible mechanisms. Maternal antibodies could cover some viral epitopes so B cells would not recognize those viral antigens and not mount the expected humoral response after infection or vaccination [70]. Nonetheless, our results indicate that high levels of homologous MDA decreased IAV levels at weaning and likely subsequent transmission during the growing period in the nursery.

Sow vaccination and MDA can protect sows and piglets from IAV clinical disease [36, 37, 39, 68, 69, 71]. Although sow vaccination is a common strategy to control IAV in BTW farms [32], IAV is still widespread in pigs. One of the major challenges to sow vaccination is the increasing IAV genetic diversity with about 16 different HA genetic clades currently circulating in US pig populations [14–17, 19–21, 25–27, 31, 72–80]. In this study, we found that homologous MDA against the circulating delta 2 H1 virus decreased delta 2 H1 IAV circulation in pigs after weaning. Since we did not sequence all viruses isolated from every pig nor did we sequence directly from every positive nasal swab, there is a possibility that we missed other viruses that might have been co-circulating in the study cohorts. Indeed, in one of the cohorts (Cohort 5a) we found partial sequences of 2 other viruses (H1 gamma (1A.3.3.3) and H3 subclade IV B) that had been historically identified in the sow farm. Why these viruses circulated at a low prevalence and did not spread throughout the entire population is uncertain. Immunity, viral spread and dominance of co-circulating viruses is puzzling and needs to be further studied in pig populations [81–85].

Our sequencing approach demonstrated the circulation of one predominant strain (H1 delta 2) in the 10 cohorts and in the BTW farm. We found no differences in the H1 antigenic sites between the delta 2 H1viruses detected in our study and the vaccine strains. Our results indicated that the HI antibodies at weaning affected IAV infection dynamics in the nursery. We considered that pigs originating from vaccinated sows had higher levels of antibodies able to cross-react with the farm specific delta 2 H1 strain and that these were, at least in part, responsible for the decrease in IAV infection. Although almost all pigs tested ELISA IAV NP positive at weaning, NP antibodies did not appear to be protective against IAV infection in the cohorts with high infection at weaning. This may be an additional indication that HI strain-specific antibodies were not present to protect pigs from IAV infection against the circulating H1 farm strain prior or at weaning, especially in the first 4 cohorts originating from non-vaccinated sows. Finally, we cannot fully discard the possibility that sow vaccination may have boosted IAV natural infection before vaccination.

Understanding IAV immunity is critical when trying to optimize the vaccination strategies currently used in swine production systems. There are still gaps in our understanding of the quality and quantity of antibodies being generated after IAV vaccination and/or natural infection. This is particularly important in pigs where we have several strains cocirculating in the same population. The complex evolution and landscape of IAV in pigs highlights the need for better vaccines and comprehensive vaccination strategies [30, 31, 80].

## Conclusions

In conclusion, we found that high levels of strain-specific hemagglutination inhibition antibodies at weaning decreased IAV infection at weaning, delayed time to become influenza-positive, decreased the number of weeks that pigs tested influenza-positive during the nursery stage, and decreased the overall estimated virus quantity during the infection period. Our results suggest that IAV infection and circulation in nursery pigs may be decreased by using adequate influenza sow vaccination protocols.

## Supporting information

S1 Table Supporting study data file. (XLSX)
